# How Having a Calling Leads to Job Crafting: A Moderated Mediation Model

**DOI:** 10.3389/fpsyg.2020.552828

**Published:** 2020-09-15

**Authors:** Po-Chien Chang, Honglei Rui, Amber Yun-Ping Lee

**Affiliations:** ^1^School of Business, Macau University of Science and Technology, Taipa, China; ^2^School of Economics and Trade, Guangxi University of Finance and Economics, Nanning, China; ^3^Department of Public Administration and Management, National University of Tainan, Tainan, Taiwan

**Keywords:** calling, job crafting, career commitment, occupational self-efficacy, job autonomy

## Abstract

This study examines the association between calling and crafting behavior by proposing a moderated mediation model. Drawing from the job crafting perspective and self-determination theory (SDT), career commitment is identified as the mediator, and occupational self-efficacy and job autonomy are identified as the moderators in the model, respectively. The authors tested the proposed relationships with an SPSS macro that utilizes a sample of 338 employees in a three-wave procedure. Results support all the hypotheses. The findings reveal calling to be significantly associated with employees’ job crafting behavior. Such a process begins with one’s career commitment and is strengthened by the level of occupational self-efficacy in the first stage as well as the level of job autonomy in the second stage, thus yielding a pattern of moderated mediation. These findings answer recent calls for an integrative examination of calling in the workplace by demonstrating that career commitment along with occupational self-efficacy and job autonomy represent key mechanisms in transferring one’s calling into job crafting behavior. As such, this study complements existing literature on the theoretical and practical implications of calling.

## Introduction

Dating back to the 16th century, the sense of a calling has held spiritual and religious significance. It has been represented as a response to God for a particular vocation, serving as an expression of one’s deepest self at work (Dik and Duffy, 2009; Rosso et al., 2010). We chose to focus on Dik and Duffy’s (2009) definition of calling for this study – regarded as one of the most influential articles in the calling literature. Dik and Duffy (2009) have conceptualized a calling as:

A calling is a transcendent summons, experienced as originating beyond the self, to approach a particular life role in a manner oriented toward demonstrating or deriving a sense of purpose or meaningfulness and that holds other-oriented values and goals as primary sources of motivation (p. 427).

This conceptualization makes the construct of a calling distinct from other similar concepts, such as intrinsic motivation and work meaningfulness (Duffy et al., 2018) and, therefore, motivates the current interest in understanding how having a calling affects one’s attitude and behavior at work.

Although research on the relationship between calling and work-related outcomes has been covered in previous literature (Duffy et al., 2018; Thompson and Bunderson, 2019), this study responds to recent calls for a more in-depth analysis of specific dependent variables that are exceptionally sensitive to differences in an employee’s sense of a calling (Thompson and Bunderson, 2019), such as job crafting. Job crafting is a positive behavior taken by employees themselves in order to face the challenges and opportunities brought about by their work (Berg et al., 2010b). Wrzesniewski and Dutton (2001) reasoned that employees perceiving a higher sense of calling are more likely to change proactively the way they exert control over their jobs in the process of experiencing work meaningfulness, especially in the context of the changing nature of their work. Such a reaction is in accordance with the concept of job crafting; thus, we emphasize the impact of having a calling at work and specifically on employees’ job crafting.

In order to gain a deeper understanding of how calling relates to job crafting, a comprehensive framework is needed to specify the relevant factors that facilitate such behavior, as well as the mechanism through which these factors exert their influence. Without delving into the mechanisms behind people developing a sense of calling, there is still a dearth of breakthroughs on this subject (Thompson and Bunderson, 2019). Previous research has demonstrated that experiencing work as a calling is positively related to individual attachment (e.g., commitment and engagement) and occupational clarity (e.g., career decidedness and career choice comfort) (Thompson and Bunderson, 2019), thus implying that the underlying psychological mechanisms of an individual’s perception of their work may play an important role in crafting their jobs. Furthermore, Duffy et al. (2017) and Wrzesniewski et al. (1997) proposed that people with a calling show higher levels of career commitment and are more willing to put effort into their work. This intrinsic work motivation is triggered by experiencing purpose and meaning in their work. As a result, Duffy et al. (2011) concluded that a higher sense of calling relates favorably to one’s commitment toward one’s career.

Guided by the self-determination theory (SDT), we further propose that the need for competence and autonomy are critical to motivation–personality integration and to those conditions that foster this mechanism (Ryan and Deci, 2000). For example, self-efficacy as an important personality trait in career decision-making has been demonstrated to be closely associated with an individual’s calling in samples of university students, artists, and musicians (Dobrow and Tosti-Kharas, 2011; Hirschi, 2011). That is, calling and domain-specific self-efficacy (i.e., occupational self-efficacy) are theoretically related (Hirschi, 2012). In addition, Farr-Wharton et al. (2011) believe that people demand autonomy and control in their work when they have a clear goal or mission to achieve, especially when the goal is associated with a strong belief system and values. Dovetailing with the job crafting perspective, where Berg et al. (2010b) point out that the impact of calling at work could be a continuous process with many contextual factors that may enhance or limit opportunities for employees to craft their jobs, ultimately resulting in varied responses to the challenge of job crafting from employees, we posit that the function of perceived competence is important to internalize for one’s motivation, while the experience of autonomy facilitates the internalization process to actively engage in job crafting behavior. Accordingly, the present study investigates career commitment as a potential mediator and regards occupational self-efficacy and job autonomy as the moderators in order to thoroughly examine the association between calling and job crafting.

In conclusion, theoretically, we rely on job crafting perspective as well as SDT to illustrate how calling leads to job crafting. We posit that initial outcomes of calling will be delivered *via* the establishment of career commitment, and that such a process will be heightened in the first stage when employees have a higher level of occupational self-efficacy, and in the second stage when they experience higher levels of autonomy. In doing so, we intend not only to understand the impact of calling but also to provide practical applications to ensure or even strengthen the impact of calling on job crafting. Our findings thus support recent calls for an integrative examination of calling at work. Additionally, the findings align with current trends that emphasize the increasing importance of calling and job crafting for individuals.

## Theoretical Background and Hypothesis Development

### Crafting a Calling

The conceptualization of work as a calling provides a way to capture the experience of having a calling in paid employment (Duffy et al., 2018). The benefits of having a calling have been discussed in past studies in the context of an individual and organizations (Duffy et al., 2011; Afsar et al., 2019). Having a calling in the work context may drive employees to perform better at their jobs and pursue their purpose through their careers at all costs (Wrzesniewski et al., 1997). In particular, employees with a strong calling are fully aware of themselves and their career choices; their sense of having a calling is strongly related to their inner world and feelings and leads them to self-directed behavior (Hall and Chandler, 2005). In other words, these individuals are more likely to actively shape their lives at work in order to incorporate or emphasize aspects of their calling, thus driving them to take advanced actions with a future-oriented impact.

Given that this impact has been observed in the rapidly growing literature on calling, we suggest examining the relationship between calling and job crafting. Job crafting is a specific type of proactive behavior, regarded as an anticipatory action taken by employees in order to face challenges and constraints posed by their jobs (Grant and Ashford, 2008; Berg et al., 2010a). As “job crafters” employees actively shape and redefine their jobs to reflect the subjective meaning and purpose they attach to their work, which in turn helps them to cope with ongoing changes in the workplace (Wrzesniewski and Dutton, 2001). Therefore, job crafting is a continuous process that involves the active change of task and/or relational boundaries of the job and includes altering how an individual sees the job, does the job, and the way he/she interacts with others on the job (Wrzesniewski and Dutton, 2001; Petrou et al., 2012). In the ever-changing world of work, which has grown complex to the point that it is not always possible to find a fit between oneself and one’s job (Berg et al., 2010a), the pull of a calling thus serves as a strong motivation for an employee to engage in activities that match his or her needs and purpose with the opportunities and demands of the work environment (Tims et al., 2016). As a result, employees proactively seize opportunities and resources to craft their jobs and fulfill all the responsibilities and expectations that come with their work (Fried et al., 2007; Abu Bakar et al., 2018).

Fried et al. (2007) assert that employees with a calling orientation are more likely to be engaged in job crafting. Such behaviors [e.g., identifying opportunities to improve situations, challenging the status quo, or coping with stress in advance to prevent a potentially stressful event, etc. (Crant, 2000)] are not only actions that effect change but are also anticipatory and forward-looking and are all expected to be observed in those with a high sense of calling. Calling, in this sense, is considered an incentive to actively perform future-focused and mindful actions. This enables us to infer that there should be a significant and positive correlation between calling and job crafting. That is, calling-oriented individuals may find it easier to craft their jobs (Hall and Chandler, 2005; Fried et al., 2007; Lu et al., 2014). Therefore, we argue that:

Hypothesis 1: An individual’s sense of calling is positively related to job crafting.

### The Mechanism Between Calling and Job Crafting

Despite the increasing literature on calling, unanswered questions remain, thus inhibiting corresponding research from gaining momentum in mainstream literature on organizational psychology (OP) and organizational behavior (OB) (Thompson and Bunderson, 2019). Of particular concern to managers and practitioners is whether the perception of work as a calling has implications on behavioral outcomes, and if so, what should be done to ensure favorable results. We hereby adopted SDT and the job crafting perspective to elucidate the continuous process ranging from having a calling at work to taking initiative action in organizations. In order to comprehensively capture the relationship between calling and job crafting, we suggest investigating both the employee’s disposition toward and opportunity to perform job crafting, as well as task or organizational settings that are associated with the decision to behave in a proactive manner (Crant, 2000; Fried et al., 2007; Petrou et al., 2012). This enables us to illuminate how and why employees with callings are more likely to engage in job crafting.

#### The Mediating Role of Career Commitment

From the job crafting perspective, having a calling reminds employees of who they are and what they could do to fulfill their passion and purpose at work (Berg et al., 2013). When people have a calling orientation, their self-identities and occupations are closely linked, urging them to pursue a career for the realization and fulfillment of their calling (Berg et al., 2010a). Duffy et al. (2011) suggest that those who view their work as a calling report higher levels of commitment to their current and future careers, given that career commitment is characterized by the pursuit of personal goals to which they are attached and closely identify (Colarelli and Bishop, 1990). The extent to which an individual is committed to a career is reflected by his or her level of calling; in other words, individuals with a higher sense of calling may demonstrate greater certainty about their own career direction (Thompson and Bunderson, 2019). Hence, previous research has depicted a strong association between calling and career commitment in many different occupations (e.g., Duffy and Sedlacek, 2007; Bunderson and Thompson, 2009; Dobrow, 2013).

People with a calling orientation are generally more aware of their mission and goals. They also appear to take responsibility for their own career development and subjective career success (Hall and Chandler, 2005; Xie et al., 2016), leading to the development of career commitment. In fact, the role of career commitment goes beyond just being motivated to advance one’s career; moreover, it contributes to proactive career behavior such as job crafting by tying an individual’s self to the desired goals. Such long-term commitment to a career encourages employees to go beyond their immediate job responsibilities and to take the initiative in reshaping their jobs (Grant and Ashford, 2008). As argued earlier, people with a calling who actively engage in job crafting are appropriately viewed as sculptors, rather than as sculptures, especially when it comes to careers and career management behavior (Bell and Staw, 1989).

Fryer and Payne (1984) believed that when people have a strong work motivator, such as a calling, they engage in career management activities and build a commitment to their careers in such a way that proactive career behaviors subsequently occur. Duffy et al. (2011) also present the importance of career commitment as a crucial link between calling and work-related well-being. Employees strive to match their needs and abilities with the opportunities and demands of the work environment, leading to a strong commitment to their careers (Tims et al., 2016). From the perspective of career dynamics, individuals with a calling orientation are more knowledgeable about their work roles and career progress and, thus, are more willing to anticipate and create the necessary changes to fit their own sense of what the job should be in terms of their respective careers (Petrou et al., 2012; Kim and Beehr, 2017). Such a process reflects the relationship between calling and job crafting *via* career commitment, suggesting that calling may relate to job crafting through the mediating mechanism of career commitment. Therefore, we propose that:

Hypothesis 2: An individual’s career commitment mediates the relationship between calling and job crafting.

#### The First-Stage Moderating Role of Occupational Self-Efficacy

As mentioned earlier, having a calling provides a strong motivation that drives people to keep investing in their careers in pursuit of their personal goals. In this process, people with a calling are deeply engaged in their work roles and consequently are more likely to demonstrate a need to discover and identify their own strengths to align their confidence with their ability to master career-related tasks (Hall and Chandler, 2005; Peterson et al., 2009). In addition, calling–career commitment relation is personal and associated with one’s internal motivation, and therefore, we argued that such a relationship is more likely to be affected by one’s personal traits such as self-efficacy. Based on SDT, the need for competence is essential to develop intrinsic motivation in a given domain (Ryan and Deci, 2000), implying that integrating a sense of self-efficacy with a calling may facilitate a better career decision and promote career commitment. In other words, the greater people believe in their ability to achieve their calling, the more likely they are willing to continue investing in their careers (Dobrow and Heller, 2015).

Occupational self-efficacy is defined as a domain-specific assessment of an individual’s ability to believe that he or she is capable of successfully fulfilling the tasks associated with their job (Betz, 2007; Rigotti et al., 2008). Previous studies have suggested that calling and occupational self-efficacy are theoretically interrelated (Conway et al., 2015; Dobrow and Heller, 2015). For example, Dik et al. (2009) pointed out that self-efficacy is a personal attribute that is linked to the level of “presence of” and “search for” calling; Hall and Chandler (2005) believed that when people possess higher levels of confidence in their competencies, their higher sense of calling should lead to a higher level of commitment to the career they are engaged in, contributing to their psychological success. A study by Dobrow and Tosti-Kharas (2012) found that people with a higher level of calling and self-efficacy tend to ignore negative feedback and only focus on the calling domain as central to their work identities. As Hirschi (2011) and Domene (2012) suggested, the strong link between calling and self-efficacy implies that they interact with each other and in general affect a person’s work and career. When people are more confident in their competencies, such as career decision-making, or ability, skills, and knowledge in relation to their job, they are more likely to have a clear vision regarding their calling (Conway et al., 2015). That is to say, knowing what you want to do and believing that you are capable of doing it will not only contribute to job and life satisfaction but also provide a blueprint for your career and thus increase your career commitment. Therefore, we propose that:

Hypothesis 3: An individual’s occupational self-efficacy will moderate the relationship between calling and career commitment and that the relationship will be stronger under high occupational self-efficacy than under low occupational self-efficacy.

#### The Second-Stage Moderating Role of Job Autonomy

The level of autonomy a person experiences at work is an important and pervasive contextual factor and has been identified as one of the five job characteristics that provides motivating potential for an individual (Hackman and Oldham, 1980). It describes the degree in which the job provides freedom, independence, and discretion for the employee to schedule their work (Hackman and Oldham, 1980). According to SDT, it is also positively associated with motivation, job satisfaction, commitment, involvement, and performance (Aubé et al., 2007). It is believed that to link one’s career commitment with their behaviors at work (i.e., job crafting), contextual factors may play significant roles in this matter. Previous studies have demonstrated that the way in which employees think of their work environment will affect their commitment and behaviors (Brown and Leigh, 1996). From the perspective of job crafting, it emphasizes the individual motivations that promote this action and also recognizes the situational conditions that may moderate how motivation contributes to the crafting process (Wrzesniewski and Dutton, 2001). When employees enjoy autonomy at work, their sense of control over the job and the work environment also increases (Parker, 1998), encouraging them to act proactively on opportunities to change the scope of their jobs or to move toward more valued directions.

Farr-Wharton et al. (2011) believed that people demand autonomy and control in their work when they commit to their careers and have a clear goal or mission to achieve, especially when such a goal is associated with a strong belief system and values. Accordingly, organizations can help employees engage in their careers by providing the appropriate context, such as work discretion (Berg et al., 2010a). By altering their job characteristics, employees are motivated to satisfy their commitment to work based on their subjective career goals and work identity, which in turn leads to job crafting (Kim and Beehr, 2017). In addition, Crant (2000) demonstrated that job crafting is affected by both individual and contextual factors. Thus, those with higher levels of career commitment are more likely to take an active role in their approach to work when they experience a higher level of job autonomy. Job autonomy is thought to strengthen people’s commitment to their careers, and as a result, they are more likely to put more effort into their jobs; it is believed to be a significant factor in job design and associated with the decision to behave in a proactive way (Crant, 2000). This argument implicitly indicates that people’s perception of job autonomy will alter the relationship between career commitment and job crafting. That is, when those with a strong career commitment are given a higher degree of job autonomy, they are more likely to engage in proactive working attitudes and behaviors (Rawat and Nadavulakere, 2015). Therefore, we propose that:

Hypothesis 4: An individual’s perception of job autonomy will moderate the relationship between career commitment and job crafting, such that the relationship will be stronger under high job autonomy than under low job autonomy.

**FIGURE 1:**
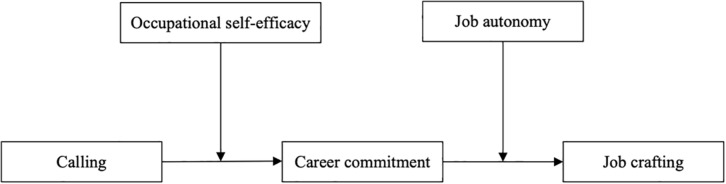
The proposed conceptual scheme.

Stemming from the argument that “enactment of a calling is the product of situational factors and an individual’s agency within a context” (Hall and Chandler, 2005, p. 167), several researchers have echoed the necessity to investigate the link between calling and personal and contextual factors (Berg et al., 2010b; Rosso et al., 2010; Duffy and Dik, 2013). In our deliberation, career commitment involves self-generated goals as well as a commitment derived from the employee’s calling, and that this will contribute to initiative and anticipatory actions. Within SDT, it is theorized that when an individual’s calling leads them to enact tasks and engage in a career that is deemed as motivational and meaningful, we firstly expect occupational self-efficacy to enhance the likelihood that the calling contributes to career commitment, with perceived autonomy as a second-stage moderator to facilitate the positive relationship between career commitment and job crafting behaviors. A higher level of occupational self-efficacy and job autonomy enables confidence in mastering career-related tasks and flexibility in work times, which alleviates possible role conflict and allows for persistence in pursuing one’s career goals (Colarelli and Bishop, 1990). It is thereby demonstrating that the level of occupational self-efficacy and job autonomy will conditionally influence the strength of the indirect relationship between a sense of calling and job crafting, as depicted in Figure 1. As a result, we reveal a pattern of first-stage and second-stage moderated indirect effect and propose an integrated moderated mediation hypothesis as below:

Hypothesis 5: The strength of the mediated relationship between calling and job crafting via career commitment varies depending on the level of occupational self-efficacy and job autonomy, such that the mediated relationship will be stronger for those with high occupational self-efficacy and with high job autonomy.

## Methods

### Participants and Procedures

The data for this study were collected from organizations in a range of different industries (e.g., service industry, manufacturing industry) operating in Guangdong province of the People’s Republic of China. In order to obtain the necessary number of participants and to facilitate valid data collection procedures, several research assistants were trained prior to the actual investigation. In addition, to avoid common method variance (CMV), a three-wave procedure was employed (Podsakoff et al., 2003), and all returned questionnaires at the different stages of the survey were coded in order to match the respondents correctly in the subsequent survey. In the first wave, 500 questionnaires were administered to employees containing measures of calling, occupational self-efficacy, and their demographic information, yielding a response rate of 84.6% (423 questionnaires). In the second wave, approximately 1 month later, we distributed 423 questionnaires to the participants who had completed the questionnaires in the first stage; 395 questionnaires were collected for career commitment and job autonomy for a 93.4% response rate. In the third wave 1 month later (i.e., 2 months from the beginning of the survey), we distributed 395 questionnaires to the participants who had completed questionnaires in both the first and second stages; 365 questionnaires were collected for job crafting for a 92.4% response rate. The reduced number of questionnaires during the second and third wave surveys was due to unexpected participant absences (e.g., business trip, sick leave). Listwise deletion of participants with missing information resulted in a final usable sample of 338 participants, representing an overall response rate of 67.6%. Participants were assured that all collected data would be utilized for research purposes only and would remain confidential. Among the included employees, 54% were male. The average age for employees was 32.79 years (SD = 7.25), and the average job tenure with a supervisor was 5.54 years (SD = 4.67).

### Measures

As the questionnaires were originally constructed in English, Chinese versions were developed based on the back-translation procedure proposed by Brislin (1980), and the data collection was implemented in a Chinese context. This was done with the help of two Chinese–English bilingual management professors in order to minimize the loss of meaning through translation. Five measures were adopted using a Likert 5-point scale, where unless specified (e.g., job crafting) ranged from 1 (strongly disagree) to 5 (strongly agree).

#### Calling

A 12-item scale developed by Dik et al. (2012) was used to measure an individual’s perception of calling in the work domain. The three dimensions and their corresponding sample items are: transcendent summons (e.g., “I believe that I have been called to my current line of work”), purposeful work (e.g., “I see my career as a path to purpose in life”), and prosocial orientation (e.g., “I am always trying to evaluate how beneficial my work is to others”). A confirmatory factor analysis (CFA) was performed to test whether the three-factor model plus an overall second-order factor would fit the data. The results showed that the fit indexes fell within an acceptable range [χ^2^ = 100.21, df = 51; comparative fit index (CFI) = 0.97; Tucker–Lewis index (TLI) = 0.96; standardized root mean square residual (SRMR) = 0.03; root mean square error of approximation (RMSEA) = 0.05], suggesting that the model would fit the data reasonably well. The Cronbach’s α for this construct was 0.90.

#### Occupational Self-Efficacy

To measure occupational self-efficacy, a six-item scale developed by Rigotti et al. (2008) was employed. A sample item was, “When I am confronted with a problem in my job, I can usually find several solutions.” The Cronbach’s α for this construct was 0.82.

#### Career Commitment

To measure career commitment, a seven-item scale developed by Blau (1988) was employed. A sample item was, “This is the ideal vocation for a life work.” The Cronbach’s α for this construct was 0.82.

#### Job Autonomy

Job autonomy was measured with a nine-item scale developed by Breaugh (1998). The three multi-item subscales focused on: (a) work method autonomy (e.g., “I am allowed to decide how to go about getting my job done”), (b) work scheduling autonomy (e.g., “I have control over the scheduling of my work”), and (c) work criteria autonomy (e.g., “I am able to modify my job objectives”). A CFA was conducted to test whether the three-factor model plus an overall second-order factor would fit the data. The results showed that the fit indexes fell within an acceptable range (χ^2^ = 80.48, df = 24; CFI = 0.96; TLI = 0.94; SRMR = 0.03), suggesting that the model would fit the data reasonably well. The Cronbach’s α for this construct was 0.90.

#### Job Crafting

Job crafting was measured using the job crafting questionnaire (JCQ) developed by Slemp and Vella-Brodrick (2013), which includes three subscales: task, relational, and cognitive crafting. The measure consists of 15 items, and participants were asked to indicate the frequency with which they had engaged in each job-crafting activity from 1 (hardly ever) to 5 (very often). Sample items included: “Introduce new approaches to improve your work” (for task crafting, five items), “Make an effort to get to know people well at work” (for relational crafting, five items), and “Remind yourself about the significance of your work for the success of the organization” (for cognitive crafting, five items). A CFA was performed to test whether the three-factor model plus an overall second-order factor would fit the data. The results showed that the fit indexes fell within an acceptable range (χ^2^ = 335.03, df = 87; CFI = 0.92; TLI = 0.91; SRMR = 0.04), suggesting that the model would fit the data reasonably well. Cronbach’s alpha for the combined scale was 0.94.

## Results

### Confirmatory Factor Analysis

A series of CFA was performed to verify the distinctiveness of the study variables. Before doing the CFA, we parceled some items based on their dimensions to control inflated measurement errors originating from multiple items for the latent variable. Thus, three item parcels for sense of calling, three item parcels for job autonomy, and three item parcels for job crafting were created. We selected five indices, including χ^2^/df, CFI, TLI, SRMR, and RMSEA, to assess the overall model fit. As shown in Table 1, the hypothesized five-factor model fits the data well [χ^2^(199) = 312.32, CFI = 0.97, TLI = 0.96, SRMR = 0.04, and RMSEA = 0.04]. Furthermore, comparing all the alternative models with the baseline model through the use of chi-square difference tests revealed that the baseline model fits the data best. Therefore, these results support the discriminability of our measures.

**TABLE 1:** The results of confirmatory factor analyses.

Measurement model	χ ^2^	df	χ ^2^/df	CFI	TLI	SRMR	RMSEA	Δχ ^2^(Δ df)
The hypothesized five-factor model	312.32	199	1.57	0.97	0.96	0.04	0.04	
Four-factor model^d^	801.75	203	3.95	0.82	0.80	0.09	0.08	489.43***(4)
Three-factor model^c^	1,418.79	206	6.89	0.63	0.59	0.13	0.13	1,106.47***(7)
Two-factor model^b^	2,338.44	208	11.24	0.36	0.29	0.17	0.17	2,026.12***(9)
One-factor model^a^	2,930.75	209	14.02	0.17	0.09	0.20	0.20	2,618.43***(10)

CFI, Comparative Fit Index; TLI, Tucker–Lewis Index; SRMR, standardized root mean square residual; RMSEA, root mean square of approximation; N = 338; ***p < 0.001. ^d^Job crafting and job autonomy combined into one factor; ^c^Job crafting, job autonomy, and career commitment combined into one factor. ^b^Job crafting, job autonomy, career commitment combined, and calling combined into one factor. ^a^All combined into one factor.

### Descriptive Statistics and Correlations

Table 2 presents the descriptive statistics and Pearson correlations for all study variables. The correlation between calling and job crafting provides initial support for our first hypothesis (*r* = 0.16, *p* < 0.01). As the demographic variables (e.g., gender, age, and job tenure with a supervisor) did not significantly correlate with the outcome variable, these were excluded as control variables.

**TABLE 2:** Means, standard deviations, and correlations among the variables.

Variables	*M*	*SD*	1	2	3	4	5	6	7
1. Gender	0.54	0.50							
2. Age	32.79	7.25	−0.01						
3. Job tenure with supervisor	5.54	4.67	−0.02	0.71***					
4. Calling	3.59	0.41	−0.07	−0.04	−0.05				
5. Occupational self-efficacy	3.73	0.47	−0.03	0.00	−0.01	0.03			
6. Career commitment	3.68	0.44	−0.07	0.02	0.07	0.16**	0.09		
7. Job autonomy	4.03	0.50	−0.03	0.08	0.10	−0.03	0.09	−0.06	
8. Job crafting	4.02	0.50	−0.06	0.08	0.02	0.16**	0.11*	0.23***	0.37***

N = 338; *p < 0.05, **p < 0.01, ***p < 0.001.

### Tests of Direct Effect and Mediation

An SPSS macro (Preacher et al., 2007; Hayes, 2018) was utilized to test the hypotheses, including direct effect, mediation, moderation, and moderated mediation models. Table 3 presents the results for Hypotheses 1 and 2. Calling was positively associated with job crafting (*B* = 0.19, *p* < 0.01), thus supporting Hypothesis 1. Next, in order to test the mediation model, the Baron and Kenny (1986) procedure was adopted for which three preconditions needed to be satisfied (i.e., the independent variable is significantly related to the dependent variable, the independent variable is significantly related to the mediator, and the mediator is significantly related to the dependent variable). As shown in Table 3, the three preconditions (i.e., Model 1, Model 2, and Model 3) were all satisfied in our study. Finally, in conducting the fourth condition, the independent variable (i.e., calling) and the mediator (i.e., career commitment) were simultaneously added into the regression model (see Table 3, Model 4). The relationship between the mediator (i.e., career commitment) and the dependent variable (i.e., job crafting) was still found to be significant (*B* = 0.24, *p* < 0.01), and the regression coefficient of the independent variable (i.e., calling) became smaller (from *B* = 0.19, *p* < 0.01 to *B* = 0.15, *p* < 0.05). In addition, to further assess the mediation, the Sobel (1982) was conducted for an indirect effect. The results revealed that the indirect path linking calling and job crafting through career commitment was significant (*z* = 2.30, *p* < 0.05). Bootstrap results confirmed the Sobel test (Table 3), with a bootstrapped 95% CI around the indirect effect not containing zero (0.01, 0.09). Thus, Hypothesis 2 was supported.

**TABLE 3:** Regression results for direct effect model and mediation model.

Variable	Model 1	Model 2	Model 3	Model 4
	X→Y	X→M	M→Y	X, M→Y
Constant	3.33***	3.08***	3.06***	2.59***
Calling	0.19**	0.17**		0.15*
Career commitment			0.26***	0.24**

**Indirect effect**				

	**Value**	***SE***	***z***	***p***

Sobel	0.04	0.02	2.30	<0.05

**Bootstrap results for indirect effect**				

	***M***	***SE***	**LL 95% CI**	**UL 95% CI**

Effect	0.04	0.02	0.01	0.09

N = 338. Unstandardized regression coefficients are reported. Bootstrap sample size = 5,000. LL, lower limit; UL, upper limit; CI, confidence interval. *p < 0.05, **p < 0.01, ***p < 0.001.

### Tests of Moderation and Moderated Mediation

**FIGURE 2:**
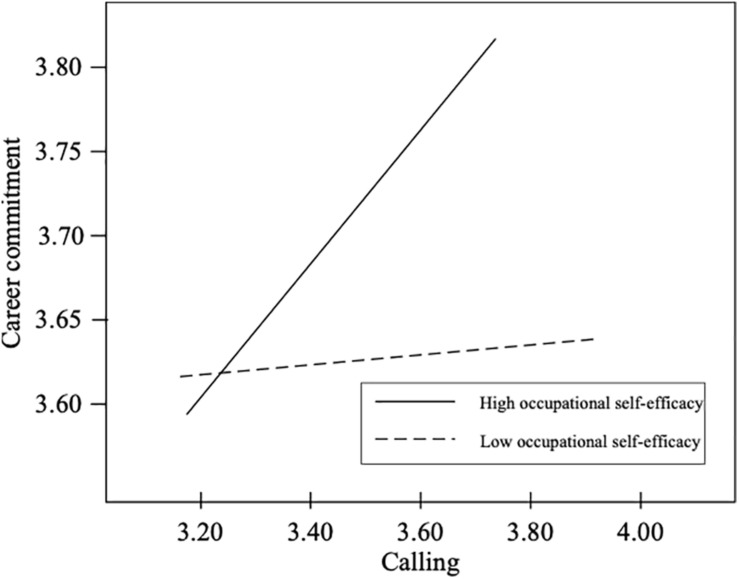
Moderating effect of occupational self-efficacy on the calling-career commitment relationship.

**FIGURE 3:**
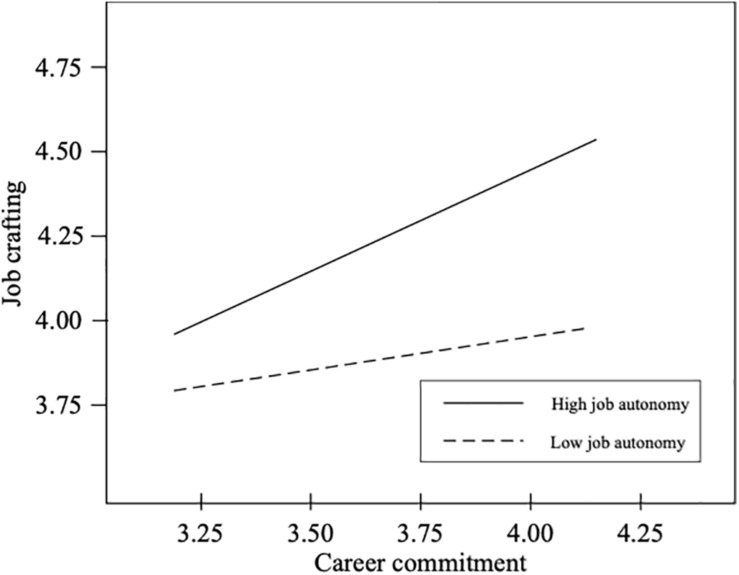
Moderating effect of job autonomy on the career commitment-job crafting relationship.

Hypothesis 3 states that occupational self-efficacy moderates the relationship between calling and career commitment in the first stage, and Hypothesis 4 states that job autonomy moderates the relationship between career commitment and job crafting in the second stage. Thus, we introduced an interaction effect between calling and occupational self-efficacy to predict career commitment and an interaction effect between career commitment and job autonomy to predict job crafting. Table 4 presents unstandardized estimates of the model for Hypothesis 3 and Hypothesis 4. The results shown in Table 4 reveal that the interaction term (calling × occupational self-efficacy) was significantly related to career commitment (*B* = 0.33, *p* < 0.01), and the interaction term (career commitment × job autonomy) was significantly related to job crafting (*B* = 0.42, *p* < 0.001). Therefore, Hypothesis 3 and Hypothesis 4 were supported. To identify the interaction effect clearly at different levels of the moderator, a conventional procedure for plotting simple slopes was applied (Figures 2, 3) at one standard deviation above and below the mean relative to occupational self-efficacy and job autonomy measure, respectively (Cohen et al., 2003). Consistent with what was expected, the slope of the relationship between calling and career commitment in the first stage was relatively strong when occupational self-efficacy was higher (simple slope = 0.32, *p* < 0.001), whereas the slope was relatively weak when occupational self-efficacy was lower (simple slope = 0.01, *p* = ns). The slope of the relationship between career commitment and job crafting in the second stage was relatively strong when job autonomy was higher (simple slope = 0.55, *p* < 0.001), whereas the slope was relatively weak when job autonomy was lower (simple slope = 0.13, *p* = ns). Next, the moderated mediation model was tested. Table 4 shows comparisons of conditional indirect effect of calling in different values of moderators. The indirect effect of calling on job crafting *via* career commitment was stronger when occupational self-efficacy was high and job autonomy was high (conditional indirect effect = 0.18, *p* < 0.01, 95% CI: 0.08–0.28). The indirect effects of calling were not significant for other values of the moderators. Therefore, Hypothesis 5 was supported.

**TABLE 4:** Regression results for moderation and moderated mediation model.

Predictor		*B*	*SE*	*B*	*SE*
**Moderation model**		Career commitment		Job crafting	
Constant		3.68***	0.02	4.02***	0.02
Calling		0.08	0.05		
Occupational self-efficacy		0.17**	0.06		
Calling × Occupational self-efficacy Career commitment Job autonomy Career commitment ×*Jobautonomy*		0.33**	0.12	0.41*** 0.34*** 0.42***	0.05 0.06 0.12
**Moderated mediation model**					
Job crafting	Moderator 1 OSE	Moderator 2 JA	*Conditional Indirect direct*	LL 95% CI	UL 95% CI
	Low (*−1SD)*	Low (*−1SD)*	0.00	−0.02	0.03
		High (*+1SD)*	0.01	−0.09	0.10
	High (*+1SD)*	Low (*−1SD)*	0.03	−0.02	0.09
		High (*+1SD)*	0.18	0.08	0.28

N = 338. Unstandardized regression coefficients are reported. OSE, occupational self-efficacy; JA, job autonomy. Bootstrap sample size = 5,000. LL, lower limit; UL, upper limit; CI, confidence interval. **p < 0.01; ***p < 0.001.

## Discussion

This research investigates the link between calling and job crafting for the purpose of advancing our understanding of the effect of calling, as well as the mechanism behind it. We identified career commitment as the mediator along with occupational self-efficacy and job autonomy as the first- and second-stage moderators, respectively, for the process of job crafting, where calling significantly contributes to employees’ crafting behaviors. This section summarizes the results of the study and discusses corresponding managerial suggestions in the hope of broadening the application of calling in organizations.

### Theoretical Implications

Research on calling has previously explored the various possible personal benefits, yet has not examined the underlying mechanisms, and thus limiting the development of calling in the field of organizational behavior and management. By focusing on the consequences of calling on the study of job crafting, the current investigation theoretically contributes in several ways to existing research on calling.

First, job crafting is shown to be extremely important in today’s work environment. Linking calling with job crafting not only prompts the interest of individuals and practitioners alike but also accentuates its impact in OP/OB literature. Second, by using a job-crafting lens, it is possible to elucidate how and why calling promotes crafting behaviors of employees. In particular, we reveal the mechanism of the job crafting process, from sensing a calling to committing to a career and subsequently taking initiative action. We further rely on SDT to explicate the influences of two important cues – occupational self-efficacy and job autonomy. Our findings are consistent with previous studies and show that experiencing a calling at work contributes to individuals’ positive outcomes (i.e., job crafting) *via* career commitment.

More specifically, the results demonstrate that increasing career commitment functions as a psychological resource, which encourages job crafters to take initiative efforts to seek challenges at work and find new ways to make their work more significant to themselves and to others (Esteves and Lopes, 2017). For example, one would take on additional tasks and change the ways in which tasks are performed, etc. (Tims et al., 2016; Chen et al., 2020). As such, employees associate their calling *via* career commitment to proactive behavior, that is, engage in job crafting strategies to make changes in their job based on their own initiative.

By employing a three-wave study to examine the mechanism of how calling contributes to job crafting, we empirically identified the position of career commitment in such a process. Furthermore, the positive moderating effect of occupational self-efficacy and job autonomy on the indirect relationship between calling and job crafting suggests that personal and contextual factors are important. From an individual perspective, there is strong support for calling enactment relating positively to occupational self-efficacy (Dobrow and Heller, 2015); while from an organizational perspective, perceived job autonomy should be beneficial to the successful implementation of a job-crafting strategy (Crant, 2000; Rawat and Nadavulakere, 2015), especially those regarding job redesign. A higher sense of occupational self-efficacy and job autonomy can be seen as an additional resource to support employees in fulfilling their calling when they practice their ideals and goals in their work. Our results suggest that when employees enact their respective callings with high self-efficacy, they are more likely to be committed to their careers, and when they are able to enjoy greater autonomy at work, their commitments to their careers are strengthened and they thereby perform their tasks in a much more proactive way. These findings also support the argument addressed by Duffy et al. (2019) and Rawat and Nadavulakere (2015) – that the mechanism of calling is complicated and, thus, personal and contextual factors are highly relevant.

### Practical Implications

Given the growing interest in calling and job crafting, this study has important implications for managers and practitioners. First, the role of career commitment in partially mediating calling and job crafting indicates that having a calling does not directly contribute to job crafting. Rather, it requires a psychological mechanism to link the sense of calling with what one wants to do and what needs to be done at work in order to effectively turn the calling into a positive action. As suggested by Duffy et al. (2011) and Thompson (2018), calling appears to establish proactive behaviors such as job crafting by fostering attachment to one’s career. Therefore, career management and career development have become indispensable human resources for management in an organization. In today’s work environment, where career transitions are not unexpected, organizations should be committed to promoting career management in order to match their employees’ needs in their careers (especially for those with a calling orientation) in exchange for their contribution to the organization.

Second, when a sense of calling is accompanied by a high degree of occupational self-efficacy, it encourages people to be more willing to engage and commit to their careers. It highlights the need for a more effective selection process as well as training and development programs whereby those selected and trained are more likely to achieve their career goals and experience career success. Therefore, to identify those with high occupational self-efficacy becomes a crucial task for human resources functions such as recruiting, selection and training, and development. In terms of recruitment and selection, job applicants with high self-efficacy should be identified through personality tests. In addition, providing employees with the skills and abilities to boost their confidence in the pursuit of their careers may enable them to think more clearly about their respective callings and careers. The relevant practices of human resource development such as career-related mentoring support and occupational skill training programs should be introduced to enhance employees’ occupational self-efficacy (Higgins et al., 2008).

And third, the significant moderating effect of job autonomy will remind managers and practitioners of the importance of job design on employees’ attitude and behavior. Although the concept of career commitment is relatively personal, our results suggest that providing employees with a higher level of autonomy at work will encourage them to engage in active and anticipatory behaviors, ultimately contributing to employees’ crafting behaviors. Wrzesniewski and Dutton (2001) have pointed out that job crafting occurs in an individual’s prescribed job, and the contextual factors determine the perceived opportunities and restrictions that an individual may experience in order to craft his or her job. In this sense, job autonomy is seen as beneficial for creating a good work environment in the organization, which may in turn generate a sense of belonging and dependence in employees. For example, higher levels of job autonomy mean more freedom to choose one’s own course of actions within a formal occupational role. It also creates opportunities for an individual to commit to a career that could answer his or her calling by enjoying higher levels of job autonomy (Berg et al., 2010b). Thus, employees will use these resources to craft their jobs in order to respond to their calling and career commitment.

### Future Directions and Limitations

Although the present study has several desirable features, the findings of our study need to be interpreted in light of its limitations. First, although empirical results support the proposed moderated mediation model, the results also suggest that future research should consider developing and testing more extensive models. For example, the partial support of the mediator reflects that there may be other mediating mechanisms. Therefore, researchers may consider the addition of other mediator variables that are recognized as predictors of job crafting (Belschak and Den Hartog, 2010) and may be affected by calling (Dobrow and Heller, 2015; Praskova et al., 2015). In addition, the present conceptual scheme only considers the individual-level moderator variable. However, as Lee et al. (2018) argue, an individual’s sense of calling exerts a positive influence on their behavior and will vary depending on their working environment (e.g., organizational climate). Therefore, future research may consider other moderating variables (such as a supportive climate and an empowerment climate), which are recognized as positive and may be critical to encouraging those with callings to conduct behaviors that organizations prefer. Second, we obtained access to the participating firms using personal contacts and the snowball approach. The inability to use random sampling may have affected the representativeness of the sample and the statistical generalization validity. It is recommended that future studies address this concern. Finally, given that this study was conducted within a Chinese context among participants from limited industry categories, we caution against generalizing the current findings to other settings. To ascertain the generalizability of the results obtained in this study, future studies should attempt to replicate our design in different cultures and in different industrial settings.

## Conclusion

Our study of calling integrates SDT with a job-crafting perspective to provide a comprehensive analysis of how calling contributes to job crafting. As such, it complements the existing literature on the wider implications of calling at work in the fields of OP and OB. We conclude that the sense of calling at work is a concept that cannot be ignored for organizations and individuals alike. When employees experience a higher sense of calling, it is easier to establish their own career commitments and subsequently demonstrate crafting behaviors in their current jobs. Such a process, through an enhancement of individual’s self-efficacy and promotion of perceived job autonomy in work design, further strengthens the positive relation between calling and job crafting.

## Data Availability Statement

The raw data supporting the conclusions of this article will be made available by the authors, without undue reservation.

## Ethics Statement

Ethical review and approval was not required for the study on human participants in accordance with the local legislation and institutional requirements. The patients/participants provided their written informed consent to participate in this study.

## Author Contributions

P-CC contributed to research idea, data analysis, and writing. AL contributed to research idea and theoretical construction. HR contributed to data collection and analysis. All authors contributed to the article and approved the submitted version.

## Conflict of Interest

The authors declare that the research was conducted in the absence of any commercial or financial relationships that could be construed as a potential conflict of interest.
